# Current role of endoscopic ultrasound for gastrointestinal and abdominal tuberculosis

**DOI:** 10.1002/jgh3.12823

**Published:** 2022-09-30

**Authors:** Hasan Maulahela, Achmad Fauzi, Kaka Renaldi, Qorina P Srisantoso, Amirah Jasmine

**Affiliations:** ^1^ Division of Gastroenterology, Pancreatobiliary, and Digestive Endoscopy, Internal Medicine Department Faculty of Medicine Universitas Indonesia‐Cipto Mangunkusumo National Central General Hospital Jakarta Indonesia; ^2^ Faculty of Medicine University of Indonesia Jakarta Indonesia

**Keywords:** abdominal tuberculosis, endoscopic ultrasound, endoscopic ultrasound fine‐needle aspiration, endoscopic ultrasound fine‐needle biopsy

## Abstract

A high incidence of tuberculosis (TB), especially in endemic countries, makes this infectious disease a concern. Abdominal TB contributes to 10% of extrapulmonary TB. Due to nonspecific clinical, radiological, and endoscopic findings, diagnosing abdominal TB continues to be a challenge. Hence, a precise diagnosis is needed. The diagnosis of gastrointestinal disease using endoscopic ultrasound (EUS) is often performed due to its high resolution and ability to provide a real‐time visual representation of the gastrointestinal tract and extramural structures. EUS‐guided fine‐needle aspiration (FNA) and fine‐needle biopsy (FNB) have helped diagnose TB as they offer an adequate specimen for cytology or histopathological examination. This method is considered safer, more effective, and more efficient. The capacity of EUS to diagnose abdominal TB based on the affected organs was examined via a literature search. We reviewed the role of EUS in diagnosing esophageal, gastric, pancreatic, peripancreatic, hepatosplenic, peritoneal, and intestinal TB. Generally, EUS aids in diagnosing abdominal TB. In some organs, it is superior to other diagnostic modalities. However, further examinations, such as cytology or histopathology and microbial, are still needed. We also studied the roles of EUS‐FNA and EUS‐FNB. EUS‐FNA has shown a high diagnostic yield in esophageal (94.3–100%), pancreatic and peripancreatic (76.2%), and intestinal TB (84.1%). As minimally invasive methods, EUS‐FNA and EUS‐FNB can successfully provide sufficient samples. EUS is a functional diagnostic modality for abdominal TB. EUS‐FNA and EUS‐FNB provide sufficient samples safely and efficiently for further cytology, histopathology, and microbial examinations.

## Graphical abstract

Gastrointestinal and abdominal tuberculosis (TB) manifestations are similar to those of other gastrointestinal diseases. Due to the non‐specific findings, diagnosing them remains a challenge. This article aims to review the role of endoscopic ultrasound (EUS) and EUS‐guided fine‐needle aspiration/fine‐needle biopsy in diagnosing gastrointestinal and abdominal TB so that patients can get appropriate treatment with a minimally invasive diagnostic approach.

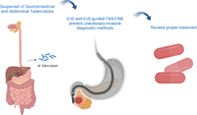

## Introduction

Tuberculosis (TB) is one of the most common infectious diseases with approximately 9.9 million cases globally.^1^ Its most frequent presentation is pulmonary TB, nevertheless; 10–20% of TB infections are extrapulmonary. Abdominal TB represents 10% of extrapulmonary TB infections, with peritoneal and intestinal TB being ubiquitous.^2^, ^3^ Patients may present with chronic diarrhea, weight loss, fever, and ascites. TB is known as “the great imitator” due to its nonspecific presentation. Therefore, diagnosing abdominal TB may be challenging due to its nonspecific findings.^3^ Precise diagnosis is vital, as different treatment approaches depend on the diagnosis.^4^, ^5^

## Endoscopic ultrasound in diagnosing abdominal tuberculosis

In recent years, the use of endoscopic ultrasound (EUS) has increased because it provides a real‐time image with enhanced resolution of the gastrointestinal tract and the extramural structures surrounding it. It is an effective, efficient, and economical method for diagnosing benign and malignant gastrointestinal diseases. A radial echoendoscope can achieve a 360° field of view with a clear visualization of each subepithelial wall. It can also identify the particular layer where the lesion arises.^6^ EUS‐guided fine‐needle aspiration (FNA) was reported to provide a suitable specimen for diagnosis in over 90% of cases. It also has the advantage of being able to reach isolated lesions. Therefore, conventional invasive methods can be avoided when obtaining histological samples.^7^ Current studies have reported the use of EUS in cases of gastrointestinal TB. A review was done by Bansal *et al*. about EUS for gastrointestinal tuberculosis. They summarized that EUS is an essential tool for diagnosing many forms of abdominal TB. EUS is also able to give a characteristic morphology and an adequate tissue sample with high sensitivity.^8^ This review article discusses the function of EUS in diagnosing abdominal TB based on the affected organs.

## Esophageal tuberculosis

Esophageal TB is uncommon and has nonspecific clinical, laboratory, radiological, and endoscopic features. With prevalence below 0.2% in all TB patients, esophageal TB usually occurs secondary to mediastinal lymphadenopathy, which causes narrowing or infiltration of the esophageal wall.^9^, ^10^ Patients may complain of systemic TB symptoms such as dysphagia, odynophagia, and retrosternal pain. Owing to the nonspecific nature of the disease, it is often mistaken for esophageal carcinoma and submucosal tumors.^11^ Differential diagnoses for esophageal tuberculosis are carcinoma of the esophagus, Crohn's disease of the esophagus, and sarcoidosis.^12^, ^13^, ^14^, ^15^ Table 1 provides distinctive characteristics of differential diagnoses.

**Table 1:** Differential diagnosis of esophageal tuberculosis (TB)

	Common symptoms	common site	Histopathology findings	Radiology findings	Endoscopy findings
Esophageal TB	Dysphagia, odynophagia, anorexia weight loss, fever^16^	Thoracic esophagus^16^	Well‐defined epithelioid granuloma with or without caseation and/or Ziehl–Neelsen (ZN) stain positive for acid‐fast bacilli (AFB)^16^	Compression, fistula^17^	Ulcers, fistula^16^
Carcinoma of esophagus	Dysphagia, anorexia, weight loss, history of GI bleeding^18^	Mid esophagus^18^	Squamous cell carcinoma (SCC), adenocarcinoma (AC)^18^	Irregular short‐segment stricture with proximal shouldering, polypoidal intraluminal filling defect, irregular mucosal ulcers^19^	Reflux esophagitis, barret's esophagus, peptic stricture^20^
Crohn's disease of the esophagus	Heartburn, regurgitation, chest pain, dysphagia, odynophagia, vomiting, weight loss^21^	Distal esophagus^21^, ^22^	Active chronic inflammation and ulcer^22^	Aphthous ulcers^21^	Not specific, friability, erosions, aphthoid ulcers, superficial or deep ulcers, nodularity and stiffness of the mucosa, cobblestone, inflammation^21^
Sarcoidosis	Dysphagia, weight loss, abdominal pain, odynophagia, hoarseness of voice/dysphonia, anemia^23^	Lower esophagus^24^	T lymphocytes, mononuclear phagocytes, and noncaseating granulomas^23^	Narrowing of the esophagus^25^	Ulcer and stricture^20^, ^26^

Esophageal TB on EUS is characterized by masses with homogenous or heterogeneous hypoechoic thickening of the esophageal wall and disruption of the esophageal adventitia. A retrospective study by Xiong *et al*. reported that among 11 patients examined with EUS, 8 presented with heterogeneous hypoechoic lesions with unclear margins and unequal interior hyperechogenic strands and foci. They observed an interrupted five‐layer structure of the esophageal wall in the other three patients. Enlarged mediastinal lymph nodes were observed in seven patients, identified by multiple rounds or an oval‐shaped enlarged hypoechogenic mass around the esophagus. EUS was also able to visualize lymph infiltration into the esophageal wall.^10^ The presence of enlarged lymph nodes may further suggest esophageal TB, as reported in previous papers.^9^, ^10^, ^16^, ^27^ EUS also plays a role in diagnosing esophageal TB cases with inconclusive pathological examination results. Nine cases of pathologically undiagnosed endoscopic TB were included in the study. All patients (100%) had EUS findings of thickening and an indefinite esophageal wall structure with partial interruption of esophageal adventitia. They observed enlarged paraesophageal lymph nodes in 88.9% of patients and calcification in esophageal hypoechoic lesions or paraesophageal lymph nodes in 44.4%. All patients received anti‐TB therapy, with observed recovery or improvement. EUS was found to be a useful diagnostic modality in this study.^28^

EUS findings in esophageal TB are classified according to stages of lymph node involvement. For Type I, the EUS finding of a lymph node is hypoechoic and homogenous, the border is distinct, and the esophageal wall might be compressed, but the adventitia is intact. For type II, the lymph node is hypoechoic and heterogenous, the borders are fused with each other, matted, and indistinct, and the esophageal wall's adventitia is breached. The five‐layer structure may be lost, and the wall might be incrassated. For type III, the lymph node is hypoechoic with anechoic areas within. The EUS findings of the border and esophageal wall are the same as for type II. For type IV, the lymph node is hypoechoic and there are also hyperechoic strands and foci with or without shadowing. The border might have peripheral calcification, and the esophageal wall findings are just like type II and type III.^29^

Compared to upper endoscopy, EUS provides more diagnostic information. EUS can precisely determine the affected layer of the esophageal wall. There has been a case of an unusual endoscopic feature where an endoscopic lesion is suggestive of angioma as it is visually bulging with a smooth surface and has a blue‐black color with a distinct border in the middle of the esophagus. It is an uncommon presentation, as esophageal TB usually shows an ulcer, sinus, or fistula as morphologic figures. In this case, EUS revealed a homogenous hypoechoic lesion with disrupted esophageal layers. Histopathology results displayed dispersed pigmentation and some epithelioid granulomas with Langhans cells and central caseous necrosis, with a background of chronic inflammatory cells. Several acid‐fast bacilli (AFB) were detected. Therefore, the patient was diagnosed with primary esophageal TB.^30^

EUS‐FNA has helped remarkably with obtaining multiple and deep submucosal biopsies with no adverse events and provides a high diagnostic yield, estimated to be 94.3–100%.^9^, ^31^, ^32^ In a study by Puri *et al*., EUS‐FNA reportedly contributed to 72% of the diagnoses of esophageal TB cases. The lymph node FNA sample was positive for AFB stain in 59.32% of cases. In comparison, endoscopic biopsy provided a diagnosis in 66% of patients, and AFB staining was positive in 16.66% of cases.^31^ It is stated that EUS‐guided fine needle aspiration cytology (FNAC) yield is better than endoscopic biopsy by 5.2%. Other studies also concluded that the yield of EUS‐FNAC was 100% compared to endoscopic biopsy (61.1%).^31^, ^33^ There have been reports on the function of EUS‐FNA in diagnosing esophageal TB when an endoscopic biopsy is inconclusive. Hence, EUS‐FNA was performed. EUS‐FNA of the caseous material of the esophagus revealed epithelioid cell granuloma with giant cell and caseation necrosis and was positive for AFB stain.^34^ A previous study by Rana *et al*. included five patients with abnormal esophageal findings that showed that endoscopic mucosal biopsy was nondiagnostic. However, this study did not compare the endoscopic mucosal biopsies performed with EUS‐FNA. Instead, EUS‐FNA was performed for mediastinal lymph node samples and successfully established a diagnosis in all patients included in the study.^9^ For mediastinal lymph node involvement, EUS‐FNA has relatively high sensitivity and specificity, with the former being 86% and the latter 100%, and positive and negative predictive values of 100% and 91%, respectively.^35^ Reports on the adverse effects of EUS‐FNA in cases of esophageal TB are unavailable.^9^, ^10^, ^16^

## Gastric tuberculosis

Gastric manifestations of TB infection are rare, with an estimated prevalence of 0.5%, and commonly occur due to secondary spread from pulmonary disease. It is classified into four types: isolated gastric TB, gastric TB secondary to pulmonary TB, gastric TB involving other parts of the gastrointestinal tract, and gastric TB in patients with HIV. Some aspects may play a role in the low incidence of gastric TB, such as the lack of gastric mucosal lymphoid tissue, rapid gastric emptying, acidic environment, and integrity of the gastric mucosa. Only 50% of patients with gastric TB are diagnosed accurately, as it is often not considered in the diagnosis.^36^

Diagnosing gastric TB using EUS alone can be challenging, considering that the presentation is similar to that of malignancy. An example of a gastric TB case suspected of malignancy was reported by Yaita *et al*. in a 60‐year‐old asymptomatic patient with a gastric lesion resembling a depressed‐type early gastric cancer, detected through esophagoduodenoscopy during a medical checkup. A localized hypoechoic lesion was found in the deep and superficial mucosa on EUS. TB infection was confirmed through biopsy based on the presence of AFB and positive polymerase chain reaction (PCR) results.^37^ Currently, no reports have directly stated the sensitivity and specificity of EUS for diagnosing gastric TB.

Gastric TB usually presents as an ulcerating or protruding lesion, with or without gastric outlet obstruction. However, there has been one case report of a gastric TB abscess. EUS revealed a heterogeneous mass arising from the submucosa of the fundus with small anechoic spaces. The presence of AFB was confirmed through FNA.^38^ EUS findings are sometimes nonspecific; hence, EUS‐guided FNA is required to establish a definitive diagnosis of gastric TB.^37^ Endoscopic biopsy has a poor yield as the predominant location of the lesion is submucosal, and endoscopic biopsy often fails to include the submucosa. EUS is considered an excellent modality for obtaining cytological samples compared with endoscopic biopsy.^39^

## Pancreatic and peripancreatic tuberculosis

Another unusual form of gastrointestinal TB is pancreatic and peripancreatic tuberculosis (PPT). In two large autopsy series of TB patients, we noticed that the percentages of pancreatic involvement were 0% and 4.7%.^40^ The transmission route of PPT infections is not yet fully understood. Several mechanisms, such as hematogenous dissemination, lymphatic dissemination, or direct spread from another location, have been proposed as possible mechanisms of infection. Patients may present with nonspecific clinical symptoms. The most commonly reported symptoms are epigastric pain, fever, and weight loss.^41^ Symptoms of pancreatic TB are similar to those of pancreatic masses. When patients are suspected of having a pancreatic mass, a differential diagnosis of pancreatic TB should be considered, especially in countries endemic to TB.^42^

Over 75% of patients with PPT show isolated pancreatic and peripancreatic masses during abdominal ultrasound or CT, which are mistaken for malignancy. Radiological findings of abdominal ultrasound, CT, or EUS show multicystic pancreatic masses usually found in the head of the pancreas. These are not distinctive characteristics of pancreatic TB and PPT, as pancreatic carcinoma may present with similar imaging findings.^43^

EUS is considered a valuable diagnostic modality for pancreatic TB. It can provide the appearance of lymphadenopathy, ductal dilatation, calcifications, and vascular invasion, in addition to assessing pancreatic lesions and size. Although PPT is rare, studies on PPT and EUS, among other types of gastrointestinal TB, are more common, as shown in Table 2. EUS findings suggestive of pancreatic TB reveal solid or mixed/solid cystic lesions in the pancreas along with a hypoechoic and anechoic echotexture without vascular invasion. An earlier study has reported that EUS‐FNA correctly diagnoses PPT in 76.2% of patients.^45^ The use of EUS has also provided the opportunity to obtain the materials needed for cytological and microbiological evaluations. Detailed screening for TB and EUS‐FNA of the pancreatic lesion is required to confirm the possibility of *Mycobacterium tuberculosis* (MTB) infection. Granulomas are a common finding on histological/cytological examination and are suggestive of TB infection. In contrast to AFB, the sensitivity rate of the PCR assay to detect MTB is higher by a margin of roughly 20–40%.^45^

**Table 2:** EUS, cytological, and microbial findings of pancreatic and peripancreatic tuberculosis

	Study type	EUS findings	Cytological diagnosis	AFB	Culture	PCR	Complications	ATT
Chakraborty *et al*.^44^	Case series (5 cases)	Hypoechoic (3/5), hypoechoic with internal hyperechoic foci (1/5), hypoechoic with septations (1/5) *Location* Head and uncinate process (1/5), head and body (1/5), head (1/5), body (2/5) *Lymph node involvement* Two cases	Epithelioid granuloma with caseation (2/5), epithelioid granuloma with caseation necrosis (1/5), epithelioid granuloma with Langhans giant cells (1/5), caseating epithelioid granuloma (1/5)	Positive (1/5), negative (4/5)	N/A	Positive in 1 case, not tested in 4 cases	N/A	Improvement (5/5)
Puri *et al*.^42^	Case series (19 cases)	Hypoechoic with anechoic areas and without vascular invasion	Caseating granuloma (19/19)	Positive (9/19)	*M. tuberculosis* (8/19)	N/A	0 case	Improvement (19/19)
Rao *et al*.^43^	Case series (2 cases of EUS‐FNA out of 14 cases studied)	Hypoechoic mass located at the head of the pancreas (2)	Suggestive of pancreatic tuberculosis (2)	Positive (2/2)	N/A	PCR positive in 8/10 cases, no details on EUS‐FNA cases	N/A	Improvement (2/2)
Song *et al*.^45^	Case series (21 cases)	Well‐demarcated hypoechoic mass (20/21)	Granulomatous inflammation (13/21 cases)	Positive (4/15)	*M. tuberculosis* (3/8)	10/15	0 case	Improvement (21/21)
Maulahela *et al*.^46^	Case report	Peripancreatic multiple lymph node enlargements	Chronic granulomatous inflammation with caseating necrosis	N/A	N/A	N/A	N/A	Improvement
Holiat *et al*.^47^	Case report	Hypoechoic lesion	Necrotizing suppurative granulomatous inflammation	Positive	*M. tuberculosis*	Positive	N/A	Lost to follow‐up
Arai *et al*.^48^	Case report	Peripancreatic hypoechoic heterogenous lesion	Necrotic tissues without malignant cells or Langhans giant cells	Negative	N/A	Positive	N/A	Improvement
Vafa *et al*.^49^	Case series (5 cases)	Heterogenous, poorly vascularized mass within the pancreatic head (1)	Granulomatous inflammation (5/5)	Positive (1/5), negative (4/5)	*M. tuberculosis* (4)	Negative (1/5), not documented (4/5)	N/A	Improvement (4/5), ongoing (1/5)
Zhu *et al*.^50^	Case report	Hypoechoic mass at duodenal bulb with heterogeneous echo, lack of unity and coherence at the duodenal wall, lesion closely adjacent to the serosa	N/A (laparotomy was done to obtain samples due to a large number of intra‐abdominal dissemination)	N/A	N/A	N/A	N/A	N/A
Chatterjee *et al*.^51^	Case series (3 cases)	Hypoechoic lymph nodes (2 cases), homogenous lymph nodes (1 case)	Granuloma (3/3)	Negative (3/3)	*M. tuberculosis* (2/3)	N/A	N/A	Improvement
Bhurwal *et al*.^52^	Case report	Heterogenous hyperechoic pancreatic lesion	Granulomatous inflammation	Positive	*M. tuberculosis*	N/A	N/A	Improvement
Dong *et al*.^53^, ^†^	Case series (12 cases)	EUS: circumscript echo‐free areas with less sharply demarcated margin and peripancreatic pseudocyst (common) CE‐EUS: hyperenhancement (performed in 3 cases) EUS‐elastography: stiffness depending on the stage of tuberculosis (performed in 5 cases)	N/A	N/A	N/A	N/A	N/A	N/A

† Used contrast‐enhanced endoscopic ultrasound (CE‐EUS).

AFB, acid‐fast bacilli; ATT, anti‐tubercular treatment; EUS, endoscopic ultrasound; PCR, polymerase chain reaction.

In the past, percutaneous FNA was commonly used. However, it has a low success rate of 50%.^45^ A previous case report indicated the importance of EUS‐FNA in diagnosing pancreatic TB. Patients with complaints of chronic epigastric pain, nausea, vomiting, weight loss, and CT findings suggestive of pancreatic malignancy underwent EUS‐FNA examinations. EUS findings showed multiple lymph node enlargements in the hilus of the spleen and the tail and body of the pancreas. Cytological results revealed chronic granulomatous inflammation with caseating necrosis, suggestive of TB infection. The patient was administered anti‐TB drugs for 9 months and showed improvement.^46^ A systematic review by Panic *et al*. stated that of 166 patients included in the analysis, 27.7% were diagnosed with EUS while 21.1% underwent EUS‐FNA.^40^ Therefore, unnecessary explorative laparotomy or pancreatic resection was avoided, and the patient was able to receive precise treatment.^41^, ^43^

Contrast‐enhanced EUS is an advancement in EUS that allows masses to be characterized and compared with the pancreatic parenchyma. Hyperenhancement has been observed in patients with pancreatic TB. EUS elastography in cases of pancreatic TB showed stiffer tissue than the pancreatic parenchyma. Stiffness depends on the stage of TB. Elastography has helped differentiate pancreatic TB from adenocarcinoma through the demarcated lesion characteristics observed in pancreatic TB. However, the elastography findings of pancreatic TB were similar to those of autoimmune pancreatitis. Therefore, EUS elastography alone may not accurately diagnose pancreatic TB.^53^

## Hepatosplenic tuberculosis

Hepatosplenic TB is a rare manifestation of gastrointestinal TB and frequently spreads either from the primary site of infection or locally from the gastrointestinal tract. Hepatic TB occurred in approximately 1% of all active TB cases. The clinical features are nonspecific. Contrast‐enhanced abdominal CT or triple‐phase liver CT is the optimal diagnostic modality for hepatic TB.^54^ Instead of reports on EUS as a diagnostic modality for hepatic TB, we found a study on EUS‐guided tuberculous liver abscess drainage. The suggested procedure for liver abscesses is surgical or percutaneous drainage. Nonetheless, a 17–32% mortality rate has been reported for surgical drainage. Recently, EUS‐guided liver abscess drainage has been reported to be a safe option for surgical or percutaneous drainage.

A previous study reported a patient with EUS findings of a hypoechoic mass‐like lesion with a central echo‐free space in the bulb of the duodenum. EUS‐guided abscess drainage was performed using a 19‐gauge needle with Doppler ultrasonography to avoid intervening blood vessels. No side effects were reported after the procedure; nevertheless, a CT scan evaluation was performed, which showed an increase in the size of another abscess. This procedure was repeated through the body of the stomach into an abscess of the left lobe. Microbial culture revealed MTB growth. The patient went home with an internal stent in place and was treated with anti‐TB therapy for 12 months. After 6 months, there was no evidence of abscess recurrence.^55^ Compared to percutaneous drainage, EUS‐guided drainage for hepatic abscess has the advantages: (i) clear visualization of the abscess cavity and landmarks in the left lobe; (ii) direct passage of the needle into the cavity; (iii) safer procedure, as the use of color Doppler helps to avoid puncturing vessels; and (iv) avoidance of transcutaneous infection. However, limitations include (i) problems in the maneuverability of long and rigid tips of the echoendoscope; (ii) linear‐array scanning limiting visibility, especially in the duodenum; and (iii) not feasible for right hepatic lobe abscess. Therefore, although some disadvantages still exist, EUS‐guided abscess drainage should be considered for abscesses inaccessible using conventional methods.^55^, ^56^

In terms of splenic TB, we found an isolated case of splenic TB diagnosed using EUS. Hypoechoic lesions in the spleen were observed on EUS. FNA revealed epithelioid granulomas with Langhans giant cells surrounding the area of caseation in the center, suggestive of TB. EUS is considered a safe and effective tool for FNA in splenic lesions that cannot be diagnosed with ultrasound or CT‐guided FNA.^57^

## Biliary tuberculosis

There have been very few reports on the use of EUS in diagnosing biliary TB. We found one study where EUS was used to eliminate the diagnosis of residual biliary microlithiasis and non‐oncologic pathology. The patient presented with jaundice and received human immunodeficiency virus and TB treatment. The patient had a history of cholecystectomy for cholelithiasis. Peculiarities in the liver on abdominal ultrasound were not detected, and neither biliary dilatation nor filling defects. The patient was presumed to have hepatitis due to TB drugs; therefore, the treatment was discontinued. Instead of showing any improvement, a progressive increase in the indirect bilirubin level was detected. An EUS was then performed, which revealed a mass along the distal common bile duct close to the duodenal papilla. The EUS‐FNA results revealed the presence of lymphocytes, that were positive for TB bacilli. The patient underwent endoscopic cholangiopancreatography with sphincterotomy, and placement of an endoprosthesis for biliary drainage and TB drugs was restarted. After 9 months of treatment, no masses were detected.^58^

## Peritoneal tuberculosis

MTB growth on ascitic fluid or peritoneal culture is the gold standard for diagnosing peritoneal TB.^59^ However, the sensitivity of ascitic fluid culture is fairly low, <50%. Laparoscopic followed by histopathological examination has high sensitivity (93%) and specificity (98%). However, it is an invasive procedure, which increases complications, especially in patients with comorbidities. Ultrasound‐ or CT‐guided imaging has been documented to be more secure and cost‐efficient. EUS may be more advanced in obtaining samples, as it can direct a biopsy needle into lesions that may be significantly too small to be identified by CT or MRI. Percutaneous biopsy may be challenging when the lesions are enveloped too well by the surrounding vascular structures.^60^, ^61^ Thus, EUS‐guided FNA may be the solution in these cases.

Peritoneal TB is classified into three categories: wet ascitic type, dry plastic type, and fibrotic fixed type. For the wet ascitic type, there is no omental thickening. For the dry plastic type, there is omental thickening and infiltration, and for the fibrotic fixed type, there is omental thickening and masses. There is also research about the mixed type where omental thickening is one of the findings.^62^ A study done by Daswani *et al*. about peritoneal tuberculosis stated that there was a thickened omentum with ascites detected by EUS. They took an EUS‐FNA sample from the thickened omentum. All of the patients' FNA cytology had granuloma with multinucleated cells, while 40% of them showed a positive AFB stain.^60^

A prior study on peritoneal TB in patients with cirrhosis revealed low ascitic fluid sensitivity and difficulty obtaining tissue for peritoneal biopsy. EUS‐FNA is a newer and safer technique for diagnosing peritoneal TB. In a study involving five patients with chronic liver disease and suspected peritoneal TB, EUS revealed a thickened omentum with ascites. Transgastric FNA via the anterior wall of the stomach was performed using a 22‐G needle, and all patients had high lymphocyte counts and cytopathological results suggestive of granuloma. However, only two of the five patients were positive for AFB. All patients were administered anti‐TB therapy, and improvement was observed. No complications were observed in this study.^60^

A study of 12 patients stated that one‐third of the cases of unexplained ascites was diagnosed with peritoneal TB. Peritoneal deposits were identified by hyperechoic rounded lesions in comparison with the surrounding anechoic ascitic fluid during EUS examination. The benefit of making smaller peritoneal and omental implants can be obviously seen in the presence of anechoic ascites in diagnosing peritoneal TB with EUS. Four patients underwent cytological examination of inflammatory cells in the absence of granulation. PCR results for MTB were positive in half of the patients. Patients also had mediastinal lymph nodes seen during the withdrawal of the echoendoscope into the esophagus. EUS‐FNA sampling showed granulomatous inflammation in all four patients, and two out of four had positive AFB stains (50%). The technical obstacle in performing FNA on small peritoneal nodules may have led to the absence of granulomas in the samples. In addition, half of these patients had portal hypertension and collateral vessels, causing the FNA procedure to be performed only in the most easily approachable peritoneal nodule, avoiding deeper punctures. This study revealed the absence of complications after the procedure, confirming EUS‐FNA as a safe, minimally invasive, and effective alternative for tissue diagnosis in patients with ascites, especially those with ambivalent diagnoses.^63^

Another case of diagnostic uncertainty has been reported by Rana *et al*. In the case of a patient with ascites and alcohol‐related cirrhosis, abdominal ultrasound was suggestive of chronic liver disease with ascites. Ascitic fluid culture and PCR analysis for MTB were performed, and the results came out negative. EUS revealed ascites and peritoneal deposits, which were identified as hyperechoic rounded lesions. A subcarinal lymph node was detected during the withdrawal of the echoendoscope. An EUS‐FNA of the lymph node was performed. Peritoneal deposits were examined cytologically, and inflammatory cells were observed. The PCR results were positive for MTB. Epithelioid cell granulomas were identified in lymph node FNA samples. The patient was then treated with antitubercular therapy and showed improvement. It can be concluded that tubercular peritonitis becomes more difficult in patients with cirrhotic ascites.^64^ Moreover, the commonly used diagnosis method for tubercular ascites may show a negative result, causing a delay in the patient's therapy.

Fever, peritonitis, and pain are possible complications of EUS paracentesis and FNA. In addition, a report revealed hypertensive emergency and pancreatitis after EUS‐paracentesis or FNA examination. However, reports on complications are infrequent.^59^ Therefore, EUS‐FNA is considered a secure method for diagnosing peritoneal TB.

## Intestinal tuberculosis

Intestinal TB and Crohn's disease are difficult to differentiate based on clinical, radiological, and endoscopic findings. In both diseases, patients may develop complaints of abdominal pain, fever, weight loss, or anemia. The most common lesions in intestinal TB are ulcerative, hypertrophic, or ulcerohypertrophic, and stricturing. Any area of the intestinal tract can be affected. The most commonly affected area is the ileocecal region (60–70% of cases).^65^, ^66^

Infection occurs when swallowed sputum containing TB bacilli invades the ileocecum, where lymph tissues are located. Invasion and inflammation occur, resulting in the thickening of the mucosal layer. Findings of intestinal TB during an endoscopic ultrasound include thickening of the mucosa, indicated by the echo level of the mucosa being hyperechoic or slightly higher than the medium level, and visible layer borders. However, no thickening of the submucosal layer was observed. This may be due to the thinness of the submucosal layer or occasional interruption due to inflammation and scarring. This finding may help distinguish intestinal TB from Crohn's disease or other diseases. However, it is not entirely specific to intestinal TB, as it is also seen in other diseases such as radiation‐induced bowel injury, solitary ulcers, and ulcers after surgery. The use of EUS in diagnosing intestinal TB has reached 84.1%, with a sensitivity of 78.3% and a specificity of 84.6%.^67^

## EUS‐guided FNA and fine‐needle biopsy in abdominal TB

EUS‐guided FNA may be effective in diagnosing gastrointestinal tuberculosis when no lymphadenopathy or solid organ involvement is seen.^4^ Needles in 19‐, 22‐, and 25‐gauge sizes are most commonly used for FNA.^6^

A previous study examined the role of EUS‐guided FNA in diagnosing abdominal lymphadenopathy. The success rate of adequate sampling of EUS‐guided FNA was 95%. A sensitivity of 75% and specificity, positive predictive value, and negative predictive value of 100% in diagnosing TB lymphadenopathy was observed in this study.^7^ TB tests such as cytology, Gene Xpert, AFB smear, and conventional culture of samples obtained from EUS‐FNA of mediastinal and intra‐abdominal lymph nodes were performed. Samples were obtained using the fanning technique, with 5–10 to and fro movements. A 10‐mL syringe attached to the needle was used to aspirate the material. Negative suction and very slow withdrawal of the needle were applied. It turns out that the Gene Xpert test had the highest sensitivity (97%) and the lowest false‐negative rate (3%), followed by cytology (sensitivity of 77% and false‐negative rate of 23%), AFB smear (sensitivity of 39% and false‐negative rate of 61%), and conventional culture (sensitivity of 3% and false‐negative rate of 87%). The Gene Xpert test also has the advantage of being able to determine rifampicin resistance. It can be concluded that EUS‐FNA followed by the Gene Xpert test is a useful tool for detecting MTB and determining drug susceptibility.^68^

Aside from the high success rate and specificity of EUS‐guided FNA, the low rate of complications is also one of the benefits of EUS‐guided FNA. Before EUS was commonly used, cytological samples were obtained through percutaneous US‐/CT‐guided FNA. However, there have been regular reports of complications, such as hematuria, pancreatitis, pneumothorax, and the need for a laparotomy.^69^ One study revealed hepatic tuberculous abscess after EUS‐FNA for abdominal lymphadenopathy. Although uncommon, the possibility of this rare complication of EUS‐FNA should be considered.^70^

Identifying and taking samples from solid lesions is usually the role of fine‐needle biopsy (FNB). Comparatively, FNB provides stromal architecture information, whereas FNA only provides a cytological specimen with little cellularity and inadequate cellular architecture.^6^ There have been limited reports on the use of EUS‐FNB in diagnosing gastrointestinal TB. Here, we report a case of intra‐abdominal tuberculous lymphadenitis. FNB was performed using a 22‐gauge ProCore needle placed in the stomach. One needle pass, with one‐to‐and‐fro needle movement, was sufficient to obtain adequate tissue material. A pathological examination revealed caseous necrotic material and AFB was identified. The patient was also positive for TB on PCR testing.^71^ EUS‐FNA examination often requires the presence of a cytopathologist or pathologist to confirm adequate tissue sampling. An EUS‐FNB examination may be advantageous in cases where there is no on‐site cytopathology assistance. It may also reduce the number of needle punctures, which also raises the possibility of obtaining sufficient specimens compared to that of EUS‐FNA.^72^

## Conclusion

The manifestation of gastrointestinal TB is often similar to that of other gastrointestinal diseases. EUS and EUS‐guided FNA/FNB may prevent unnecessary invasive diagnostic methods for gastrointestinal TB. Therefore, more patients can be precisely diagnosed and receive proper treatment using a less invasive procedure.

## References

[1] World Health Organization . Global tuberculosis report; 2021.

[2] Mor P , Dahiya B , Parshad S , Gulati P , Mehta PK . Recent updates in diagnosis of abdominal tuberculosis with emphasis on nucleic acid amplification tests. Expert Rev. Gastroenterol. Hepatol. 2022; 16: 33–49. 34923892 10.1080/17474124.2022.2021068

[3] Abu‐Zidan FM , Sheek‐Hussein M . Diagnosis of abdominal tuberculosis: lessons learned over 30 years: pectoral assay. World J. Emerg. Surg. 2019; 14: 1–7. 31338118 10.1186/s13017-019-0252-3 PMC6626328

[4] Yavuz A , Toksöz Yıldırım AN , Akan K , Tuncer İ . A never‐ending challenge: intestinal tuberculosis or inflammatory bowel disease. Cureus. 2021; 13: 1–6. 10.7759/cureus.16282 PMC8346276 34373825

[5] Merino‐Gallego E , Gallardo‐Sánchez F , Gallego‐Rojo FJ . Intestinal tuberculosis and Crohn's disease: The importance and difficulty of a differential diagnosis. Rev. Esp. de Enferm. Dig. 2018; 110: 650–6. 10.17235/reed.2018.5184/2017 30168341

[6] Sooklal S , Chahal P . Endoscopic ultrasound. Surg. Clin. N. Am. 2020; 100: 1133–50. 33128884 10.1016/j.suc.2020.07.003

[7] Pausawasdi N , Maipang K , Sriprayoon T , Charatchareonwitthaya P . Role of endoscopic ultrasound‐guided fine‐needle aspiration in the evaluation of abdominal lymphadenopathy of unknown etiology. Clin. Endosc. 2022; 55: 1–8. 34974679 10.5946/ce.2021.218-IDEN PMC8995993

[8] Bansal RK , Kaur G , Choudhary NS , Puri R . Endoscopic ultrasound for gastrointestinal tuberculosis. Gsyt. In: Sharma, V. (eds). *Tuberculosis of the Gastrointestinal system*. Singapore: Springer; 2022.

[9] Rana SS , Bhasin DK , Rao C , Srinivasan R , Singh K . Tuberculosis presenting as dysphagia: clinical, endoscopic, radiological and endosonographic features. Endosc. Ultrasound. 2013; 2: 92–5. 24949371 10.4103/2303-9027.117693 PMC4062249

[10] Xiong J , Guo W , Guo Y , Gong L , Liu S . Clinical and endoscopic features of esophageal tuberculosis: a 20‐year retrospective study. Scand. J. Gastroenterol. 2020; 55: 1200–4. 32881605 10.1080/00365521.2020.1813799

[11] Danna BJ , Harvey AW , Woc‐Colburn LE . Esophageal tuberculosis—a mass of confusion. Am. J. Med. 2020; 133: e589–90. 32272102 10.1016/j.amjmed.2020.02.038

[12] Khanna V , Kumar A , Alexander N , Surendran P . A case report on esophageal tuberculosis—a rare entity. Int. J. Surg. Case Rep. 2017; 35: 41–3. 28437671 10.1016/j.ijscr.2017.03.042 PMC5403801

[13] Chowdhury MS . Oesophageal tuberculosis mimicking oesophageal carcinoma—a case report. 2016. Available from URL: https://www.researchgate.net/publication/300999955.

[14] Griga T , Duchna HW , Orth M *et al*. Tuberculous involvement of the oesophagus with oesophagobroncheal fistula. Dig. Liver Dis. 2002; 34: 528–31. 12236488 10.1016/s1590-8658(02)80113-x

[15] Calaras D , Munteanu O , Botnaru V . Sarcoidosis and tuberculosis: a rare combination? Eur. Respir. J. 2012; 40: P3635.

[16] Dahale AS , Kumar A , Srivastava S , Varakanahalli S , Sachdeva S , Puri AS . Esophageal tuberculosis: uncommon of common. JGH Open. 2018; 2: 34–8. 30483561 10.1002/jgh3.12043 PMC6207044

[17] Tirumani H , Rosenthal MH , Tirumani SH , Shinagare AB , Krajewski KM , Ramaiya NH . Esophageal carcinoma: current concepts in the role of imaging in staging and management. Can. Assoc. Radiol. J. 2015; 66: 130–9. 25770628 10.1016/j.carj.2014.08.006

[18] Choksi D , Kolhe K , Ingle M *et al*. Esophageal carcinoma: an epidemiological analysis and study of the time trends over the last 20 years from a single center in India. J. Fam. Med. Prim. Care. 2020; 9: 1695–9. 10.4103/jfmpc.jfmpc_1111_19 PMC7266213 32509674

[19] Itskoviz D , Tamary H , Krasnov T *et al*. Endoscopic findings and esophageal cancer incidence among fanconi anemia patients participating in an endoscopic surveillance program. Dig. Liver Dis. 2019; 51: 242–6. 30249500 10.1016/j.dld.2018.08.010

[20] Abraham A , Hajar R , Virdi R , Singh J , Mustacchia P . Esophageal sarcoidosis: a review of cases and an update. ISRN Gastroenterol. 2013; 5: 1–9. 10.1155/2013/836203 PMC3603204 23533794

[21] Pimentel Andréa M , Rocha R , Santana GO . Crohn's disease in upper gastrointestinal tract. World J. Gastrointest. Pharmacol. Ther. 2019; 10: 35–49. 30891327 10.4292/wjgpt.v10.i2.35 PMC6422852

[22] Anton G , Decker G , Loftus E v , Pasha TM , Tremaine WJ , Sandborn WJ . Crohn's disease of the esophagus: clinical features and outcomes. Inflamm Bowel Dis. 2001; 7: 113–9. 11383583 10.1097/00054725-200105000-00006

[23] Ni B , Lu X , Gong Q *et al*. Surgical outcome of esophageal tuberculosis secondary to mediastinal lymphadenitis in adults: experience from single center in China. J. Thorac. Dis. 2013; 5: 498–505. 23991308 10.3978/j.issn.2072-1439.2013.08.33 PMC3755661

[24] Albertson MJ . *Sarcoidosis presenting as dysphagia*. Proceedings of UCLA Healthcare; 2015.

[25] Yelamanchi R , Chukka GK , Manda GDT , Dokania M . Sarcoidosis: a rare cause of dysphagia. Indian J. Surg. 2021; 83: 1–2.

[26] Szatkowski J , Cassano H . *Esophageal sarcoidosis*. Proceedings of UCLA Health; 2020;24.

[27] Kaur G , Bakshi P , Verma K , Kumar M . Esophageal tuberculosis: EUS FNA diagnosis of uncommon presentation as a cystic lesion. Diagn. Cytopathol. 2011; 40: 352–4. 21538949 10.1002/dc.21654

[28] Zhu R , Bai Y , Zhou Y *et al*. EUS in the diagnosis of pathologically undiagnosed esophageal tuberculosis. BMC Gastroenterol. 2020; 20: 291. 32859167 10.1186/s12876-020-01432-7 PMC7455903

[29] Dahale AS , Kumar A , Srivastava S . In: Sharma V (ed.). Tuberculosis of the Gastrointestinal System. Singapore: Springer, 2022; 47.

[30] Li YX , Nian WD , Wang HH . A case of esophageal tuberculosis with unusual endoscopic feature. Clin. Res. Hepatol. Gastroenterol. 2018; 42: e5–6. 28578936 10.1016/j.clinre.2017.04.010

[31] Puri R , Khaliq A , Kumar M , Sud R , Vasdev N . Esophageal tuberculosis: role of endoscopic ultrasound in diagnosis. Dis. Esophagus. 2012; 25: 102–6. 21777339 10.1111/j.1442-2050.2011.01223.x

[32] Tang Y , Shi W , Sun X , Xi W . Endoscopic ultrasound in diagnosis of esophageal tuberculosis: 10‐year experience at a tertiary care center. Dis. Esophagus. 2017; 30: 1–6. 10.1093/dote/dox031 28575247

[33] Birda CL , Kumar A , Gupta P , Singh H , Sharma V . Oesophageal tuberculosis: a systematic review focusing on clinical management. Dysphagia. 2021; 37: 1–5. 34482490 10.1007/s00455-021-10360-x

[34] Sharma V , Rana SS , Chhabra P , Sharma R , Gupta N , Bhasin DK . Primary esophageal tuberculosis mimicking esophageal cancer with vascular involvement. Endosc. Ultrasound. 2016; 5: 61–2. 26879170 10.4103/2303-9027.175924 PMC4770626

[35] Fritscher‐Ravens A , Ghanbari A , Topalidis T *et al*. Granulomatous mediastinal adenopathy: can endoscopic ultrasound‐guided fine‐needle aspiration differentiate between tuberculosis and sarcoidosis? Endoscopy. 2011; 43: 955–61. 21833904 10.1055/s-0031-1271110

[36] Chaudhary P , Khan AQ , Lal R , Bhadana U . Gastric tuberculosis. Indian J, Tuberc. 2019; 66: 411–7. 31439189 10.1016/j.ijtb.2018.10.004

[37] Yaita H , Nakamura S , Kurahara K *et al*. Gastric tuberculosis resembling depressed type, early gastric cancer. Endoscopy. 2014; 46: E669–70. 25526418 10.1055/s-0034-1390865

[38] Nayyar E , Torres JA , Malvestutto CD . Tuberculous gastric abscess in a patient with AIDS: a rare presentation. Case Rep. Infect. Dis. 2016; 2016: 1–4. 10.1155/2016/5675036 PMC4867059 27239353

[39] Arabi NA , Musaad AM , Ahmed EE , Ibnouf MMAM , Abdelaziz MSE . Primary gastric tuberculosis presenting as gastric outlet obstruction: a case report and review of the literature. J. Med. Case Rep. 2015; 9: 4–9. 26577440 10.1186/s13256-015-0748-8 PMC4650840

[40] Panic N , Maetzel H , Bulajic M , Radovanovic M , Löhr JM . Pancreatic tuberculosis: a systematic review of symptoms, diagnosis and treatment. United Eur. Gastroenterol. J. 2020; 8: 396–402. 10.1177/2050640620902353 PMC7226685 32213022

[41] Sharma V , Rana SS , Kumar A , Bhasin DK . Pancreatic tuberculosis. J. Gastroenterol. Hepatol. 2016; 31: 310–8. 26414325 10.1111/jgh.13174

[42] Puri R , Thandassery RB , Eloubeidi MA , Sud R . Diagnosis of isolated pancreatic tuberculosis: the role of EUS‐guided FNA cytology. Gastrointest. Endosc. 2012; 75: 900–4. 22440205 10.1016/j.gie.2011.12.026

[43] Rao RN , Pandey R , Rana MK , Rai P , Gupta A . Pancreatic and peripancreatic tuberculosis presenting as hypoechoic mass and malignancy diagnosed by ultrasound‐guided fine‐needle aspiration cytology. J Cytol. 2013; 30: 130–5. 23833404 10.4103/0970-9371.112658 PMC3701338

[44] Chakraborty D , Ghosh R , Halder A , Mondal D , Misra D . Endoscopic ultrasonographic diagnosis of pancreatic tuberculosis in immunocompetent patients—a case series from eastern India. Indian J. Gastroenterol. 2021; 40: 636–40. 34528180 10.1007/s12664-021-01171-x

[45] Song TJ , Lee SS , Park DH *et al*. Yield of EUS‐guided FNA on the diagnosis of pancreatic/peripancreatic tuberculosis. Gastrointest. Endosc. 2009; 69: 484–91. 19231490 10.1016/j.gie.2008.10.007

[46] Maulahela H , Fauzi A . Peripancreatic tuberculosis lymphadenopathy: the role of endoscopic ultrasound for diagnosis. Acta Med. Indones. 2021; 53: 457–9. 35027493

[47] Hoilat GJ , Abdu M , Hoilat J , Gitto L , Bhutta AQ . A rare case of pancreatic tuberculosis diagnosed via endoscopic ultrasound‐guided fine needle aspiration and polymerase chain reaction. Cureus. 2020; 12: e8795. 32724744 10.7759/cureus.8795 PMC7381879

[48] Arai J , Kitamura K , Yamamiya A *et al*. Peripancreatic tuberculous lymphadenitis diagnosed via endoscopic ultrasound‐guided fine‐needle aspiration and polymerase chain reaction. Intern. Med. 2017; 56: 1049–52. 28458310 10.2169/internalmedicine.56.7509 PMC5478565

[49] Vafa H , Arvanitakis M , Matos C *et al*. Pancreatic tuberculosis diagnosed by EUS: one disease, many faces. J. Pancreas. 2013; 14: 256–60. 10.6092/1590-8577/1355 23669474

[50] Zhu M , Zhang N , Tao W , Wang Z , He S . Pancreatic tuberculosis with vascular involvement and peritoneal dissemination in a young man. Case Rep. Med. 2017; 2017: 1–6. 10.1155/2017/4396759 PMC5610859 29081806

[51] Chatterjee S , Schmid ML , Anderson K , Oppong KW . Tuberculosis and the pancreas: a diagnostic challenge solved by endoscopic ultrasound. A case series. J. Gastrointest. Liver Dis. 2012; 21: 7–9. 22457868

[52] Bhurwal A , Haq MM , Sapru S , Tortora M , Ramasamy D . Isolated pancreatic tuberculosis mimicking pancreatic cancer: a diagnostic challenge. Case Rep. Gastrointest. Med. 2018; 2018: 7871503. 29850296 10.1155/2018/7871503 PMC5925152

[53] Dong Y , Jürgensen C , Puri R *et al*. Ultrasound imaging features of isolated pancreatic tuberculosis. Endosc Ultrasound. 2018; 7: 119–27. 28721972 10.4103/2303-9027.210901 PMC5914183

[54] Hickey AJ , Gounder L , Moosa MYS , Drain PK . A systematic review of hepatic tuberculosis with considerations in human immunodeficiency virus co‐infection. BMC Infect. Dis. 2015; 15: 1–11. 25943103 10.1186/s12879-015-0944-6 PMC4425874

[55] Itoi T , Ang TL , Seewald S *et al*. Endoscopic ultrasonography‐guided drainage for tuberculous liver abscess drainage. Dig. Endosc. 2011; 23: 158–61. 21535224 10.1111/j.1443-1661.2011.01115.x

[56] Noh SH , Park DH , Kim YR *et al*. EUS‐guided drainage of hepatic abscesses not accessible to percutaneous drainage (with videos). Gastrointest. Endosc. 2010; 71: 1314–9. 20400078 10.1016/j.gie.2009.12.045

[57] Nasa M , Choudhary NS , Guleria M , Puri R . Isolated splenic tuberculosis diagnosed by endoscopic ultrasound‐guided fine needle aspiration. Indian J. Tuberc. 2017; 64: 134–5. 28410696 10.1016/j.ijtb.2016.01.002

[58] Schneider NC , Coelho N , Dutra P *et al*. Diagnosis of ganglionar tuberculosis by endoscopic ultrasonography. Endosc. Ultrasound. 2014; 3: S12. PMC4569907 26425509

[59] Sharma V , Rana SS , Ahmed SU , Guleria S , Sharma R , Gupta R . Endoscopic ultrasound‐guided fine‐needle aspiration from ascites and peritoneal nodules: a scoping review. Endosc. Ultrasound. 2017; 6: 382–8. 29251272 10.4103/eus.eus_96_17 PMC5752760

[60] Daswani R , Kumar A , Singla V *et al*. Endoscopic ultrasound (EUS) guided fine needle aspiration: a new modality to diagnose peritoneal tuberculosis in presence of decompensated cirrhosis—a case series and review of literature. J. Clin. Exp. Hepatol. 2018; 8: 205–9. 29892185 10.1016/j.jceh.2017.09.007 PMC5992303

[61] Koff A , Azar MM . Diagnosing peritoneal tuberculosis. BMJ Case Rep. 2020; 13: e233131. 10.1136/bcr-2019-233131 PMC7035809 32033999

[62] Agarwala R , Zubair Z . Classification and case definitions in gastrointestinal tuberculosis. In: Sharma V (ed.). Tuberculosis of the Gastrointestinal System. Singapore: Springer, 2022; 32.

[63] Rana SS , Bhasin DK , Srinivasan R , Singh K . Endoscopic ultrasound‐guided fine needle aspiration of peritoneal nodules in patients with ascites of unknown cause. Endoscopy. 2011; 43: 1010–3. 21833905 10.1055/s-0031-1271111

[64] Rana SS , Bhasin DK , Srinivisan R , Singh K . Endoscopic ultrasound fine‐needle aspiration of peritoneal deposits for diagnosis of tubercular peritonitis in a cirrhotic patient with ascites. Endoscopy. 2010; 42: 306–7. 21113884 10.1055/s-0030-1255800

[65] Goyal P , Shah J , Gupta S , Gupta P , Sharma V . Imaging in discriminating intestinal tuberculosis and Crohn's disease: past, present and the future. Expert Rev. Gastroenterol. Hepatol. 2019; 13: 995–1007. 31559871 10.1080/17474124.2019.1673730

[66] Limsrivilai J , Shreiner AB , Pongpaibul A *et al*. Meta‐analytic Bayesian model for differentiating intestinal tuberculosis from Crohn's disease. Am. J. Gastroenterol. 2017; 112: 415–27. 28045023 10.1038/ajg.2016.529 PMC5551982

[67] Qiu EQ , Guo W , Cheng TM *et al*. Diagnostic classification of endosonography for differentiating colorectal ulcerative diseases: a new statistical method. World J. Gastroenterol. 2017; 23: 8207–16. 29290657 10.3748/wjg.v23.i46.8207 PMC5739927

[68] Mohindra S , Nayak HK , Mohindra N *et al*. Diagnostic yield of endoscopic ultrasound–guided fine‐needle aspiration of tubercular lymphadenitis using combination of cytology and Gene Xpert *Mycobacterium tuberculosis*/rifampicin (MTB/RIF) genes. Indian J. Gastroenterol. 2021; 40: 630–5. 33443639 10.1007/s12664-020-01136-6

[69] Saieg MA , Yazawa F , Horta M , Rossini LG , Fracassi MTM , Del Carlo Bernardi F . The utility of endoscopic ultrasound guided fine needle aspiration in the diagnosis of infectious diseases—report of three cases. Case Rep. Infect. Dis. 2013; 2013: 1–4. 10.1155/2013/512182 PMC3872382 24392229

[70] Kumagai S , Muta Y , Yazumi S . Tuberculous abscess formation with liver invasion after endoscopic ultrasound‐guided fine‐needle aspiration for abdominal lymphadenopathy. Endoscopy. 2014; 46: 188–9. 24756288 10.1055/s-0034-1365147

[71] Lee TH , Cho JY , Bok GH , Cho WY , Jin SY . Intra‐abdominal tuberculous lymphadenitis diagnosed using an endoscopic ultrasonography‐guided procore needle biopsy. Clin. Endosc. 2013; 46: 77–80. 23423647 10.5946/ce.2013.46.1.77 PMC3572357

[72] James TW , Baron TH . A comprehensive review of endoscopic ultrasound core biopsy needles. Expert Rev. Med. Devices. 2018; 15: 127–35. 29334842 10.1080/17434440.2018.1425137

